# Keeping Patients Safe Online: A Study of Refractive Surgery Advertisements on Social Media

**DOI:** 10.7759/cureus.68643

**Published:** 2024-09-04

**Authors:** Emanuel Y Tan, Zeping Tan, Jin Han Khaw, Ryian Mohamed, Arthur Okonkwo, Harry Petrushkin, Daniel Gore, Rami Mohamed

**Affiliations:** 1 Orthopedics, Tan Tock Seng Hospital, Singapore, SGP; 2 ENT, Northwick Park Hospital, London, GBR; 3 Faculty of Life Sciences and Medicine, King's College London, London, GBR; 4 Ophthalmology, Western Eye Hospital, London, GBR; 5 Ophthalmology, Moorfields Eye Hospital, London, GBR

**Keywords:** refractive errors, use of social media in ophthalmology, marketing on social media, ethical social media use, refractive eye surgery

## Abstract

Introduction

Refractive error is the leading cause of visual impairment and blindness globally. Increasingly, patients are exposed to information about refractive surgery through social media advertisements. While national guidelines specify how refractive surgery should be advertised in traditional media, it is unclear to what extent these standards are adhered to in the emerging commercial arena of social media. The adherence of refractive surgery advertisements on social media to professional standards is poorly studied.

Method

We retrospectively analyzed the content of refractive surgery advertisements on the social media platform "TikTok," shown in the United Kingdom (UK) from October 2022 to October 2023, and compared them to the guidelines set out by The Royal College of Ophthalmologists (RCOphth) and the Advertising Standards Authority (ASA).

Results

We found that 39/51 (76%) of advertisements did not state the specific pathology to be corrected, and 41/51 (80%) did not specify a surgical procedure. Additionally, 33/51 (65%) of advertisements included at least one financial inducement, 44/51 (86%) contained misleading claims. None of the analyzed advertisements provided specific prices, offered refractive surgery as a competition prize, or featured celebrity endorsements. No medical jargon was found in any of the advertisements. The most viewed advertisement was seen by over 1.2 million unique users, with the median number of views for all advertisements being 34,000.

Conclusion

In conclusion, our analysis revealed that none of the refractive surgery advertisements on a popular social media platform met the standards set by RCOphth or ASA. This study presents the first qualitative analysis of social media refractive surgery advertisements, offering insights into what users can expect and providing recommendations for patients, doctors, social media platforms, and regulators to enhance refractive surgery advertising in the future.

## Introduction

One of the leading causes of vision impairment and blindness globally is refractive error [1]. Among refractive errors, myopia, colloquially known as short-sightedness, affects more than two billion people worldwide and is now considered a growing public health challenge [2]. It is estimated that by 2050, myopia will affect approximately five billion people [2]. In line with this epidemiological shift, the demand for surgical correction of myopia is also increasing. In 2020, 3.6 million refractive surgery procedures were undertaken globally, with projections of up to six million procedures by 2025 [3]. The refractive surgery market was valued at USD 11.1 million in 2024 and is estimated to reach USD 25.2 million by 2032, equating to a compound annual growth rate of 12% [4].

A new way by which individuals are now exposed to information about refractive surgery is through social media. As of the start of 2023, the number of social media users was equal to 84% of the total UK population [5]. Worldwide, there are an estimated 3.9 billion social media users [6]. The impact of social media is potentially greatest among younger demographics, in 2023, about 25% of social media users in the UK were aged between 18 and 29 years, and nearly 50% were aged 30 to 49 years [7].

Given the prevalence of refractive error, many social media users will likely suffer from it and be exposed to advertisements for refractive surgical correction during their social media use. Social media platforms, therefore, provide a large marketplace in a poorly regulated virtual space. These concerns are compounded because many social media users are young, likely with little to no comorbidities and have limited experience navigating healthcare systems and providers [8].

The Royal College of Ophthalmologists (RCOphth) has published advertising standards for refractive error correction procedures, including "Professional Standards for Refractive Surgery" [9] and "Refractive Surgery Advertising and Marketing Standards" [10]. However, whether refractive surgery advertisements on social media meet these professional standards is poorly studied. We provide the first qualitative analysis of social media refractive surgery advertisements, determining what users can expect and making recommendations for improvement.

## Materials and methods

We used the "Refractive Surgery Advertising and Marketing Standards" guidelines [10] set out by RCOphth to create nine domains for assessing the advertisements we encountered on social media platforms. Each domain was then validated against the ASA's nonbroadcast code (medicines, medical devices, health-related products, and beauty products) [11] for marketing communications to complete our data collection tool. The nine domains are described in Table 1.

**Table 1:** Components of domains one to nine LASIK: Laser-assisted in situ keratomileusis; LASEK: laser-assisted subepithelial keratectomy; SMILE: small incision lenticule extraction; PRK: photorefractive keratectomy; RK: radial keratotomy; AK: astigmatic keratotomy; PIOL: phakic intraocular lens; RLE: refractive lens exchange

Domain	Description
Domain one	Assesses whether any specific refractive pathology is stated, such as myopia, hyperopia, or astigmatism, to ensure that the patient is adequately informed about the expected outcome of the procedure
Domain two	Evaluates whether any specific surgical procedure, such as LASIK, LASEK, SMILE, PRK, RK, AK, PIOL, or RLE, is mentioned, allowing the patient to be informed about the purpose of the procedure and enabling them to independently research the benefits and risks of each option
Domain three	Investigates whether any financial inducements, such as time-limited offers, package deals, or other incentives, are offered, to identify potential unethical persuasion of patients to undergo unnecessary treatments
Domain four	Examines price advertising to ensure that patients are provided with sufficient information regarding the financial commitment required for the procedure
Domain five	Addresses whether refractive surgery is offered as a competition prize, as this could indicate unethical persuasion of patients to undergo unnecessary treatments
Domain six	Looks at the use of celebrity endorsements, which could suggest unethical persuasion
Domain seven	Scrutinizes any misleading claims, such as promises of permanent or immediate improvement in vision, statements suggesting no risks, or efficacy claims without supporting evidence, to ensure that patients can make informed decisions based on a realistic understanding of the procedure's risks
Domain eight	Considers the use of medical jargon, emphasizing the importance of patients fully understanding the procedure and its associated risks
Domain nine	Measures the number of unique advertisement viewers, providing insight into the impact of each advertisement analysis

**Figure 1:**
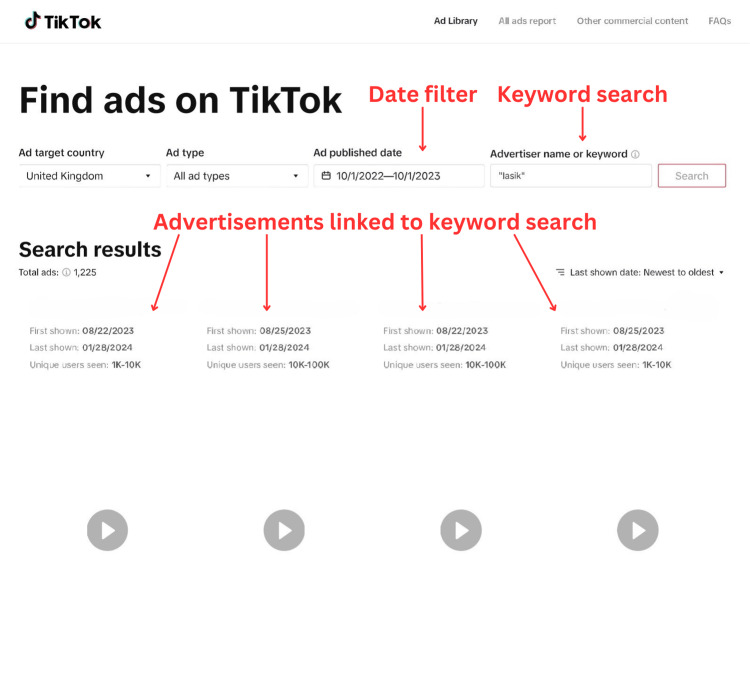
View of TikTok advertisement library annotated with relevant functions used in data gathering for this study LASIK: Laser-assisted in situ keratomileusis

**Figure 2:**
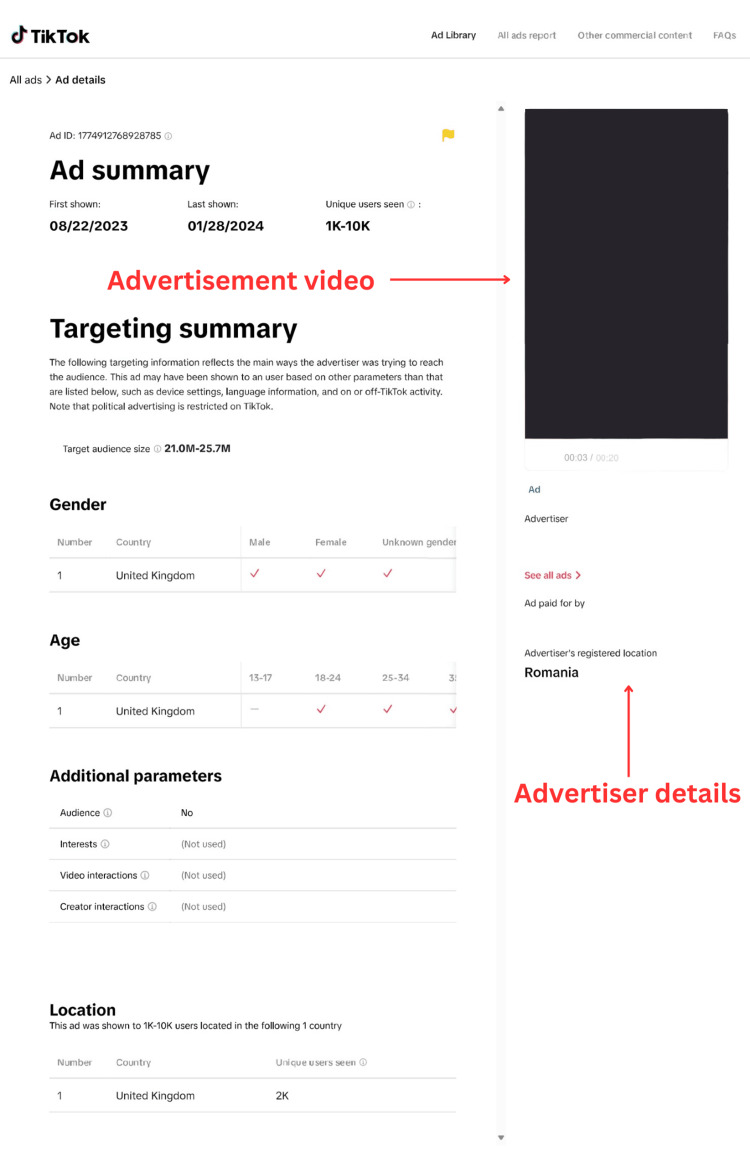
View of individual advertisement details and advertising video annotated with relevant details

We deemed the social media platform "TikTok" to be the most suitable for data collection. "TikTok" attracts a younger and therefore more vulnerable demographic and is the sixth most used social media platform in the UK [12]. Adults aged 18-34 make up over 69% of all "TikTok" adult users [13]. Additionally, the platform offers a public advertising library that indexes each advertisement by keywords (Figures 1, 2). This feature made it user-friendly, as we could narrow our search to include only refractive surgery advertisements. This was logistically advantageous, making data collection effective, time-saving, and reproducible.

Using this advertisement library, we generated a list of keywords to search for advertisements related to refractive surgery. We searched for advertisements targeting the UK from October 1, 2022, to October 1, 2023. Data prior to October 2022 was not available, possibly due to server data storage constraints or regular data clearing functions. The keywords are listed: refractive surgery, laser eye surgery, lens surgery, vision correction surgery, laser vision correction, LASIK, LASEK, SLE, PRK, RK, AK, SMILE, PIOL, laser-assisted in situ keratomileusis, laser-assisted epithelial keratomileusis, small incision lenticule extraction, photorefractive keratectomy, radial keratotomy, astigmatic keratotomy, SMILE surgery, PRK surgery, RK surgery, AK surgery, surface laser treatments, RLE surgery, and phakic interocular lens implantation.

Our keyword search yielded more than 1,000 video advertisements. After identifying and removing duplicates, we were left with 51 unique advertisements. Initially, we used the thumbnails of each advertisement video to check for duplicates. However, some advertisements had the same thumbnail but different video or audio content. Therefore, we defined a duplicate as "an advertisement that had exactly the same video and audio content as any advertisement we had previously analyzed." Each advertisement was approximately 30 seconds long, and we watched each one in full to identify keywords related to the nine domains and determine if a domain had been satisfied.

## Results

Domains one and two

**Figure 3:**
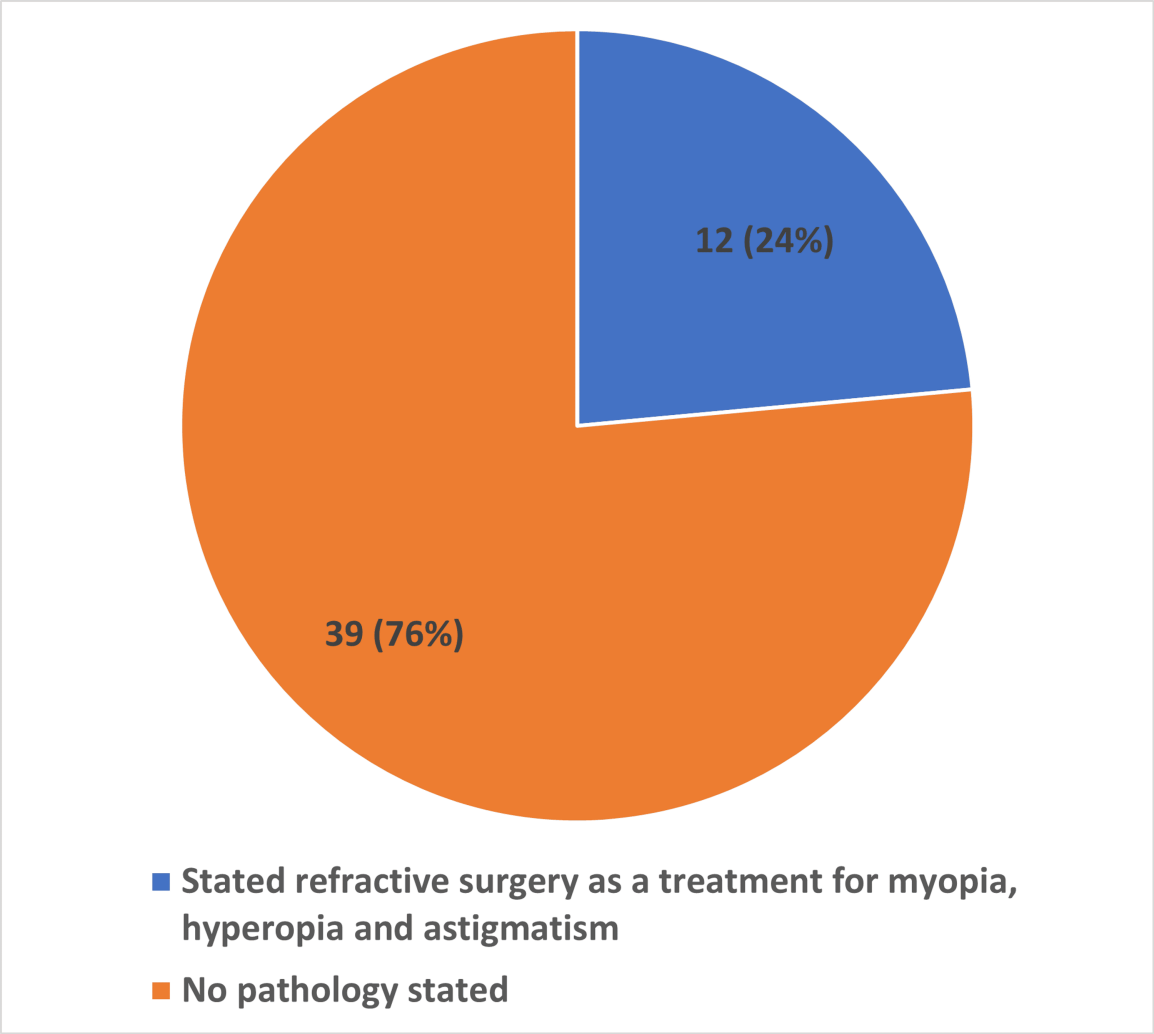
Domain one This graph shows that 12/51 (24%) of advertisements stated refractive surgery as a treatment for myopia, hyperopia, and astigmatism

**Figure 4:**
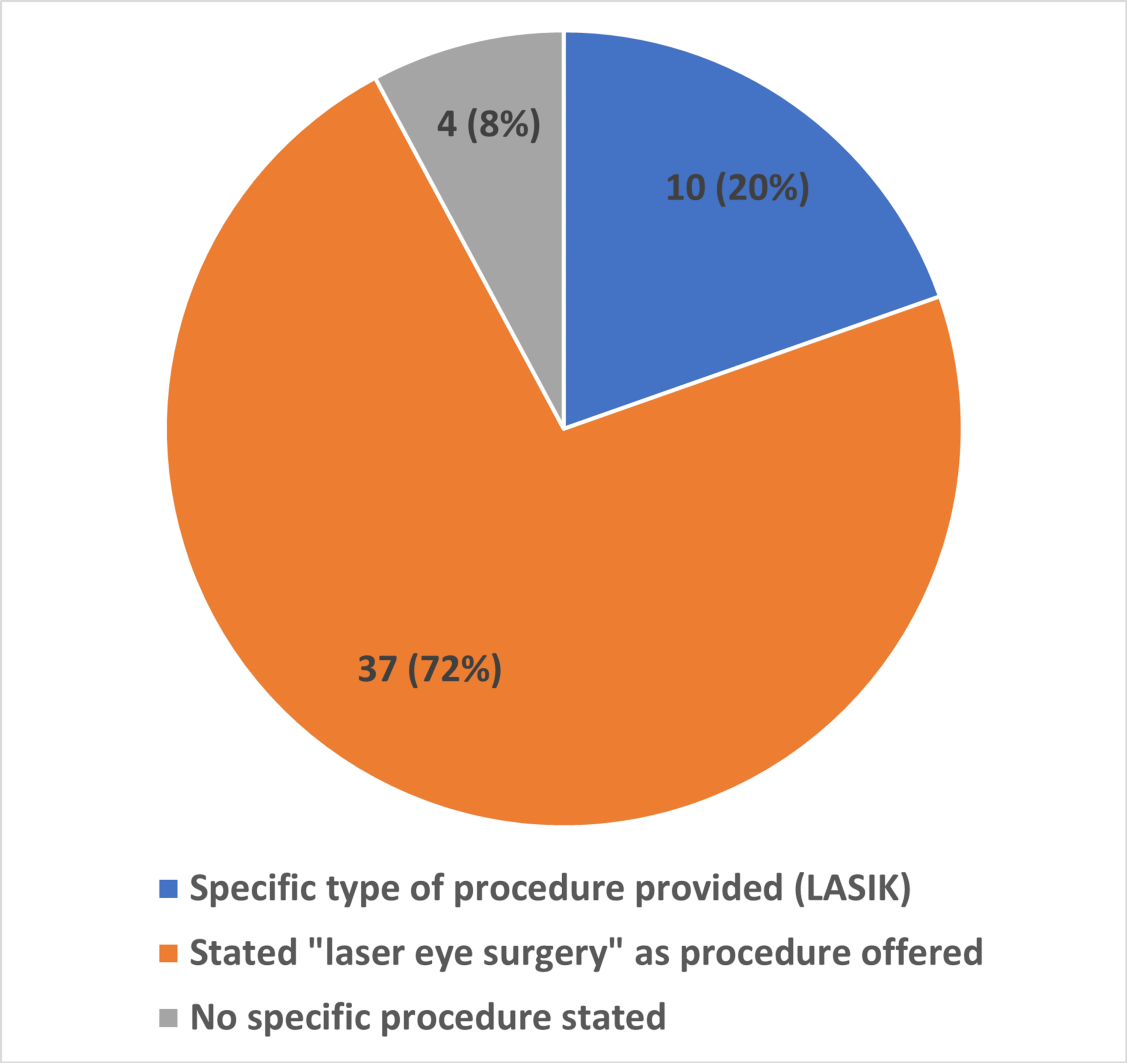
Domain two *LASIK: Laser-assisted in situ keratomileusis This graph shows that 37/51 (72%) of advertisements stated "laser eye surgery" as the procedure offered for refractive error correction, 10/51 (20%) specified LASIK as the procedure, and 4/51 (8%) did not state a specific procedure

A total of 12/51 (24%) stated refractive surgery as a treatment for myopia, hyperopia, and astigmatism. A total of 39/51 (76%) did not state a specific pathology which would be corrected or mentioned generally "vision correction" or "eyesight restoration" in the audio or video elements of the advertisement. A total of 10/51 (20%) stated laser-assisted in situ keratomileusis (LASIK) as the type of procedure provided. A total of 4/51 (8%) did not state a specific procedure which was being advertised for. Finally, 37/51 (72%) stated “laser eye surgery” as the procedure that was being offered (Figures 3, 4).

Domain three

**Figure 5:**
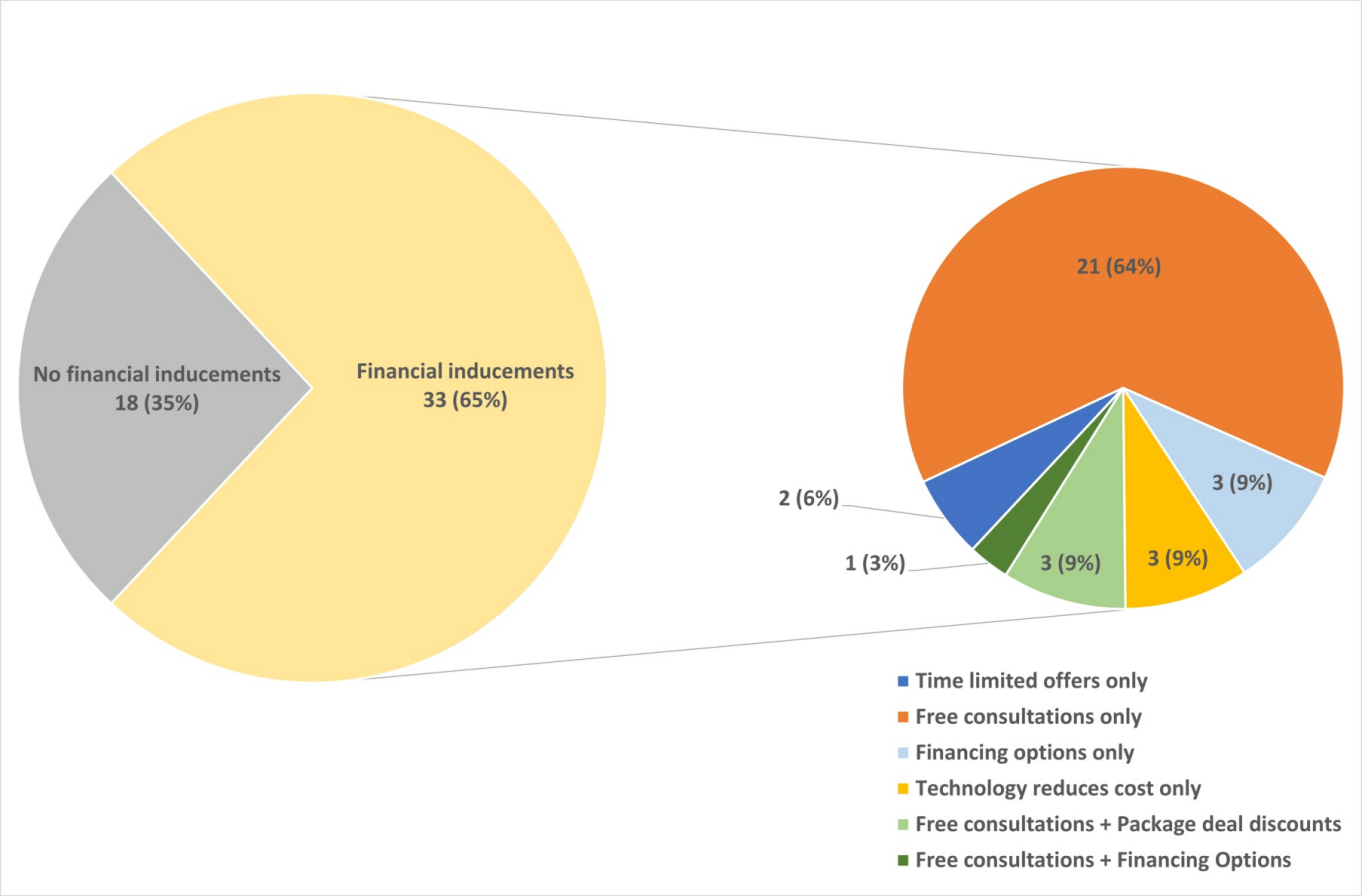
Domain three Percentage of financial inducements overall and subtypes encountered. This graph shows that 33/51 (65%) of advertisements included at least one financial inducement, of which 21/33 (64%) were free consultations only, 3/33 (9%) were financing options only, 3/33 (9%) stated technology reduced cost of surgery, 3/33 (9%) were free consultations plus package deal discounts, 2/33 (6%) were time limited offers only, and 1/33 (3%) were free consultations plus financing options.

A total of 33/51 (65%) of advertisements had at least one financial inducement. Of these 33 advertisements, 2/33 (6%) had a time limited offer of “New Year’s deals." A total of 21/33 (64%) offered only free consultations either stated in the audio or as a frame in the video, such as “free consultation worth GBP 800,” “looking for 300 people to receive a free consultation” or “born before 1983? Then, you could be eligible for a free laser eye consultation.” A total of 3/33 (9%) offered “financing options,” but it was unclear what these options were. A total of 3/33 (9%) stated the procedures were more affordable due to technology, but it was unclear how this technology contributed to the reduced costs. A total of 3/33 (9%) offered free consultations and package deal discounts. A total of 1/33 (3%) offered free consultations and financing options. Again, it was unclear what these financing options were (Figure 5).

Domains four, five, and six

None of the analyzed advertisements had specific prices advertised, and refractive surgery offered as a competition prize or celebrity endorsements.

Domain seven

**Figure 6:**
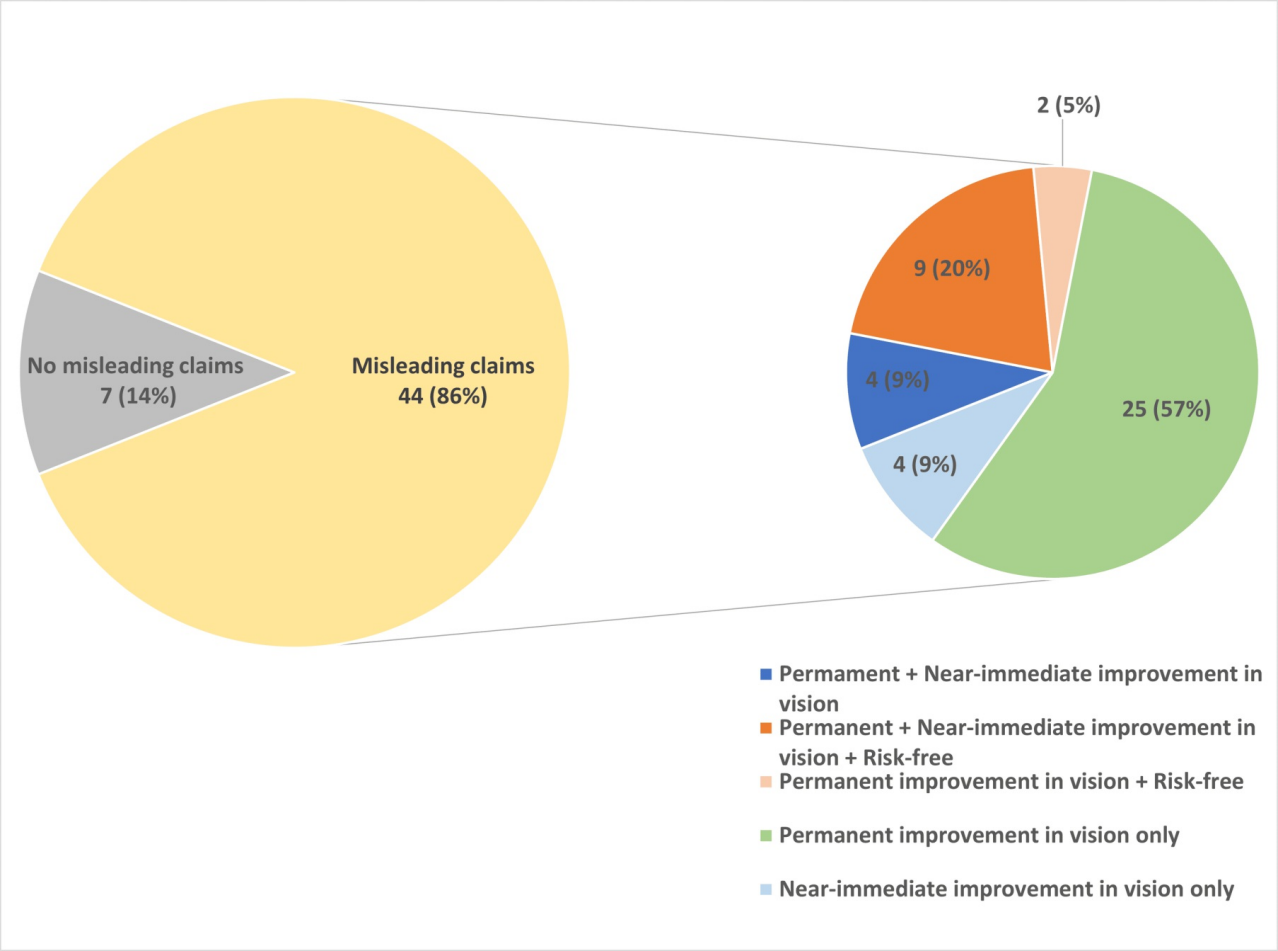
Domain seven Percentage of misleading claims overall and subtypes encountered.  This graph shows that 44/51 (86%) of advertisements included misleading claims, of which 25/44 (57%) were permanent improvement in vision only, 9/44 (20%) were permanent and near-immediate improvement in vision plus risk-free, 4/44 (9%) were permanent plus near-immediate improvement in vision, 4/44 (9%) were near-immediate improvement in vision only, and 2/44 (5%) were permanent improvement in vision plus risk-free

A total of 44/51 (86%) of advertisements had at least one claim that was potentially misleading. Of these 44 advertisements, 25/44 (57%) of advertisements only suggested a permanent improvement in vision. Statements such as “get rid of the glasses forever” were made in these advertisements. A total of 4/44 (9%) only suggested a near-immediate improvement in vision with statements such as “return to work in only 48 hours.” A total of 11/44 (25%) included information which could be interpreted as being risk-free, such as “the only question you’ll have is why I didn’t do it sooner” or “safe due to advanced technology.” We found no evidence to back up these claims made in the videos or examples of these advanced technologies. Of these 11/44 (25%) advertisements, 9/44 (20%) also included suggestions of permanent and near-immediate improvement of vision while being risk-free. The remaining 2/44 (5%) advertisements suggested a permanent improvement of vision alongside being risk-free (Figure 6).

Domain eight

No use of medical jargon was found in any of the advertisements.

Domain nine

The most viewed advertisement was seen by upward of 1.2 million unique users at the time of writing (see Table 2 for a full analysis). This number is likely to continue increasing as advertisements can be shown more than once after initial broadcast. The median number of views for all advertisements was 34,000 at the time of writing, and the total number of views was 6,393,000. One company produced 40/51 (78%) of the analyzed advertisements.

**Table 2:** Example full analysis of advertisement 49 LASIK: Laser-assisted in situ keratomileusis; LASEK: laser-assisted subepithelial keratectomy; SMILE: small incision lenticule extraction; PRK: photorefractive keratectomy; RK: radial keratotomy; AK: astigmatic keratotomy; PIOL: phakic intraocular lens; RLE: refractive lens exchange Advertisement 49 satisfied domains three ("we are looking for people for a free laser eye consultation") and seven ("If you want to get rid of the glasses forever") and had 1.2 million views at time of analysis

Domain number	Domain criteria	Aims	Advertisement 49
1	Any specific refractive pathology stated?-myopia, hyperopia, astigmatism	To ensure the patient is adequately informed on the expected outcome of the procedure	No
2	Any specific surgical procedure stated?: LASIK, LASEK, SMILE, PRK, RK, AK, PIOL, RLE	To ensure the patient is adequately informed on the purpose of the procedure and can independently research the benefits and risks of each procedure	No
3	Any financial inducements offered?: Time-limited offers, package deals (for example, buy one get one free, friends and family discounts), others	To identify any unethical persuasion of patients to undergo treatments they may not need	Yes: “We are looking for people for a free laser eye consultation”
4	Price advertising: Is there any price advertising and are the patients given enough information in the advertisement?	To ensure the patient is fully informed of the financial commitment required for the procedure	No
5	Refractive surgery offered as a competition prize?	To identify any unethical persuasion of patients to undergo treatments they may not need	No
6	Any celebrity endorsements?	To identify any unethical persuasion of patients to undergo treatments they may not need	No
7	Misleading claims: For example: permanent improvement in vision, immediate improvement in vision, no risks involved, efficacy claims with no backing evidence	To ensure patients can make an informed decision based on the risks of the procedure	Yes: “If you want to get rid of your glasses forever”
8	Use of medical jargon?	To ensure patients do not undergo a procedure without full understanding of the procedure and associated risks	No
9	Number of unique advertisement viewers?	A measure of the impact of each advertisement analyzed	1.2 million views (at time of analysis)

Advertisements 27 and 50 were removed from the TikTok library due to violations of terms of service. We chose to include both the advertisements in our results as the domain analysis was completed before removal from the platform, but we were unable to transcribe these two advertisements. See Table 3 for all data collected.

**Table 3:** Full results table *LASIK: Laser-assisted in situ keratomileusis This table includes all raw data collected from the nine assessed domains, transcripts and "TikTok" advertisement library weblinks for all 51 advertisements (excluding transcripts and view counts for advertisements 27 and 50 due to removal for terms of service violations). The view counts were updated for each advertisement on May 3, 2024

Advertisement number	1	2	3	4	5	6	7	8	9	10	11	12	13	14	15	16	17	18	19	20	21	22	23	24	25	26	27	28	29	30	31	32	33	34	35	36	37	38	39	40	41	42	43	44	45	46	47	48	49	50	51
Specific pathology stated?	FALSE	TRUE	FALSE	TRUE	FALSE	FALSE	FALSE	FALSE	TRUE	FALSE	FALSE	FALSE	FALSE	TRUE	FALSE	FALSE	FALSE	FALSE	TRUE	FALSE	TRUE	TRUE	FALSE	FALSE	FALSE	FALSE	FALSE	TRUE	TRUE	FALSE	FALSE	FALSE	TRUE	TRUE	TRUE	FALSE	FALSE	FALSE	FALSE	FALSE	FALSE	FALSE	FALSE	FALSE	FALSE	FALSE	FALSE	FALSE	FALSE	FALSE	FALSE
If pathology stated: myopia or hyperopia	None	All of the above	None	All of the above	None	None	None	None	All of the above	None	None	None	None	All of the above	None	None	None	None	All of the above	None	All of the above	All of the above	None	None	None	None	None	All of the above	All of the above	None	None	None	All of the above	All of the above	All of the above	None	None	None	None	None	None	None	None	None	None	None	None	None	None	None	None
Specific surgical procedure stated?	FALSE	FALSE	FALSE	FALSE	FALSE	FALSE	FALSE	FALSE	FALSE	FALSE	FALSE	FALSE	TRUE	FALSE	FALSE	FALSE	FALSE	TRUE	FALSE	FALSE	FALSE	FALSE	TRUE	FALSE	FALSE	FALSE	TRUE	FALSE	FALSE	FALSE	FALSE	TRUE	FALSE	FALSE	FALSE	FALSE	TRUE	TRUE	TRUE	FALSE	FALSE	TRUE	FALSE	FALSE	FALSE	FALSE	FALSE	FALSE	FALSE	FALSE	TRUE
If specific surgical procedure stated, type of surgery?	None	None	None	None	None	None	None	None	None	None	None	None	LASIK	None	None	None	None	LASIK	None	None	None	None	LASIK	None	None	None	LASIK	None	None	None	None	LASIK	None	None	None	None	LASIK	LASIK	LASIK	None	None	LASIK	None	None	None	None	None	None	None	None	LASIK
Financial Inducements included?	TRUE	TRUE	FALSE	TRUE	TRUE	FALSE	FALSE	TRUE	TRUE	TRUE	TRUE	TRUE	TRUE	TRUE	TRUE	TRUE	FALSE	TRUE	TRUE	TRUE	TRUE	TRUE	FALSE	TRUE	TRUE	TRUE	FALSE	TRUE	TRUE	TRUE	TRUE	FALSE	TRUE	TRUE	TRUE	TRUE	FALSE	FALSE	FALSE	FALSE	FALSE	FALSE	FALSE	FALSE	FALSE	TRUE	TRUE	TRUE	TRUE	FALSE	FALSE
Example of financial inducements	1. Free consultation	1. Free consultation 2. Procedure saves money (Don't have to waste money on contact lenses or glasses)		1. Financing options 2.Procedure saves money (Don't have to waste money on contact lenses or glasses ever again)	1. Free consultation	None	None	1. Free consultation	1.Financing options 2. Procedure saves money (Don't have to waste money on contact lenses or glasses)	1. Procedure saves money (Don't have to waste money on contact lenses or glasses)	1. Free consultation	1. Free consultation	1. Special discounts for New Year's 2. Procedure saves money (LASIK saves money)	1. Free consultation 2.Procedure saves money (Don't have to waste money on contact lenses or glasses)	1. Free consultation	1. Free consultation	None	1. Special discounts for New Year's	1.Procedure saves money (Don't have to waste money on contact lenses or glasses)	1. Free consultation	1. Free consultation 2.Procedure saves money (Don't have to waste money on contact lenses or glasses)	1. Free consultation	None	1. Free consultation worth 800	1. Free consultation	1. Free consultation	None	1. Financing options 2.Procedure saves money (Don't have to waste money on contact lenses or glasses)	1. Free consultation	1. Free consultation	1. Free consultation	None	1. Free consultation 2.Procedure saves money (Don't have to waste money on contact lenses or glasses) 3.Financing options	1.Procedure saves money (Don't have to waste money on contact lenses or glasses)	1. Free consultation	1. Free consultation	None	None	None	None	None	None	None	None	None	1. Free consultation	1. Free consultation	1. Free consultation	1. Free consultation	None	None
Time limited offer included?	FALSE	FALSE	FALSE	FALSE	FALSE	FALSE	FALSE	FALSE	FALSE	FALSE	FALSE	FALSE	TRUE	FALSE	FALSE	FALSE	FALSE	TRUE	FALSE	FALSE	FALSE	FALSE	FALSE	FALSE	FALSE	FALSE	FALSE	FALSE	FALSE	FALSE	FALSE	FALSE	FALSE	FALSE	FALSE	FALSE	FALSE	FALSE	FALSE	FALSE	FALSE	FALSE	FALSE	FALSE	FALSE	FALSE	FALSE	FALSE	FALSE	FALSE	FALSE
Package deals? (e.g. buy one get one free or reduced prices for friends and family)	FALSE	FALSE	FALSE	FALSE	FALSE	FALSE	FALSE	FALSE	FALSE	FALSE	FALSE	FALSE	FALSE	FALSE	FALSE	FALSE	FALSE	FALSE	FALSE	FALSE	FALSE	TRUE	FALSE	TRUE	FALSE	TRUE	FALSE	FALSE	FALSE	FALSE	FALSE	FALSE	FALSE	FALSE	FALSE	FALSE	FALSE	FALSE	FALSE	FALSE	FALSE	FALSE	FALSE	FALSE	FALSE	FALSE	FALSE	FALSE	FALSE	FALSE	FALSE
Price advertising?	FALSE	FALSE	FALSE	FALSE	FALSE	FALSE	FALSE	FALSE	FALSE	FALSE	FALSE	FALSE	FALSE	FALSE	FALSE	FALSE	FALSE	FALSE	FALSE	FALSE	FALSE	FALSE	FALSE	FALSE	FALSE	FALSE	FALSE	FALSE	FALSE	FALSE	FALSE	FALSE	FALSE	FALSE	FALSE	FALSE	FALSE	FALSE	FALSE	FALSE	FALSE	FALSE	FALSE	FALSE	FALSE	FALSE	FALSE	FALSE	FALSE	FALSE	FALSE
If yes, is the patient given sufficient information regarding the advertised price? (e.g., eligibility criteria, specific details of treatment provided)	N/A	N/A	N/A	N/A	N/A	N/A	N/A	N/A	N/A	N/A	N/A	N/A	N/A	N/A	N/A	N/A	N/A	N/A	N/A	N/A	N/A	N/A	N/A	N/A	N/A	N/A	N/A	N/A	N/A	N/A	N/A	N/A	N/A	N/A	N/A	N/A	N/A	N/A	N/A	N/A	N/A	N/A	N/A	N/A	N/A	N/A	N/A	N/A	N/A	N/A	N/A
Dishonest price advertising (cross-checking advertisement and provider website)	FALSE	FALSE	FALSE	FALSE	FALSE	FALSE	FALSE	FALSE	FALSE	FALSE	FALSE	FALSE	FALSE	FALSE	FALSE	FALSE	FALSE	FALSE	FALSE	FALSE	FALSE	FALSE	FALSE	FALSE	FALSE	FALSE	FALSE	FALSE	FALSE	FALSE	FALSE	FALSE	FALSE	FALSE	FALSE	FALSE	FALSE	FALSE	FALSE	FALSE	FALSE	FALSE	FALSE	FALSE	FALSE	FALSE	FALSE	FALSE	FALSE	FALSE	FALSE
Refractive surgery offered as a competition prize?	FALSE	FALSE	FALSE	FALSE	FALSE	FALSE	FALSE	FALSE	FALSE	FALSE	FALSE	FALSE	FALSE	FALSE	FALSE	FALSE	FALSE	FALSE	FALSE	FALSE	FALSE	FALSE	FALSE	FALSE	FALSE	FALSE	FALSE	FALSE	FALSE	FALSE	FALSE	FALSE	FALSE	FALSE	FALSE	FALSE	FALSE	FALSE	FALSE	FALSE	FALSE	FALSE	FALSE	FALSE	FALSE	FALSE	FALSE	FALSE	FALSE	FALSE	FALSE
Celebrity endorsements?	FALSE	FALSE	FALSE	FALSE	FALSE	FALSE	FALSE	FALSE	FALSE	FALSE	FALSE	FALSE	FALSE	FALSE	FALSE	FALSE	FALSE	FALSE	FALSE	FALSE	FALSE	FALSE	FALSE	FALSE	FALSE	FALSE	FALSE	FALSE	FALSE	FALSE	FALSE	FALSE	FALSE	FALSE	FALSE	FALSE	FALSE	FALSE	FALSE	FALSE	FALSE	FALSE	FALSE	FALSE	FALSE	FALSE	FALSE	FALSE	FALSE	FALSE	FALSE
Any claims that can be interpreted as misleading?	1. No glasses or contact lenses forever	1. No glasses or contact lenses forever	1. No glasses or contact lenses forever	1.Why didn't I do it sooner 2.Improvements in 48 hours	None	1.No glasses or contact lenses forever 2.Improvement in 48 hours	1.>99% effective surgery 2. Surgery took minutes	1. No glasses or contact lenses forever	1. No glasses or contact lenses forever 2. Why didn't I do it sooner 3.Improvements in 48 hours	1. No glasses or contact lenses forever	1. No glasses or contact lenses forever	1. No glasses or contact lenses forever	None	None	1. No glasses or contact lenses forever	None	1. Surgery took minutes	1. No glasses or contact lenses forever	1. No glasses or contact lenses forever	1. No glasses or contact lenses forever	1. No glasses or contact lenses forever	1. Surgery is quick, 2. No glassess or contact lenses forever	1. No glasses or contact lenses forever	1. No glasses or contact lenses forever	1. No glasses or contact lenses forever	1. No glasses or contact lenses forever	1. Surgery took minutes	1.Why didn't I do it sooner 2.Improvements in 48 hours 3. No glassess or contact lenses forever	1. Surgery is quick 2. No glasses or contact lenses forever	1. No glasses or contact lenses forever	1. No glasses or contact lenses forever	1. No glasses or contact lenses forever	1. Improvements in 48 hours 2. Why didn't I do it sooner 3. No glasses or contact lenses forever	1. Why didn't I do it sooner 2. No glasses or contact lenses forever	1. Why didn't I do it sooner 2. No glasses or contact lenses forever	1. No glasses or contact lenses forever	1. Surgery is quick 2. Surgery is reliable 3. Surgery is safe	1. Surgery is quick 2. Surgery is reliable 3. Surgery is safe	1. Surgery is quick 2. Surgery is reliable 3. Surgery is safe	None	1. Surgery is quick 2. Surgery is reliable 3. Surgery is safe	None	None	None	None	None	1. No glasses or contact lenses forever	1. No glasses or contact lenses forever	1. No glasses or contact lenses forever	None	1. Surgery is quick
"Permanent improvement in vision"	TRUE	TRUE	TRUE	TRUE	TRUE	TRUE	TRUE	TRUE	TRUE	TRUE	TRUE	TRUE	TRUE	TRUE	TRUE	FALSE	FALSE	TRUE	TRUE	TRUE	TRUE	TRUE	TRUE	TRUE	TRUE	TRUE	FALSE	TRUE	TRUE	TRUE	TRUE	TRUE	TRUE	TRUE	TRUE	TRUE	TRUE	TRUE	TRUE	FALSE	TRUE	FALSE	FALSE	FALSE	FALSE	FALSE	TRUE	TRUE	TRUE	FALSE	FALSE
"Immediate or near-immediate improvement in vision"	FALSE	FALSE	FALSE	TRUE	FALSE	TRUE	TRUE	FALSE	TRUE	FALSE	FALSE	FALSE	FALSE	FALSE	FALSE	FALSE	TRUE	FALSE	FALSE	FALSE	FALSE	TRUE	FALSE	FALSE	FALSE	FALSE	TRUE	TRUE	TRUE	FALSE	FALSE	TRUE	TRUE	FALSE	FALSE	FALSE	TRUE	TRUE	TRUE	FALSE	TRUE	FALSE	FALSE	FALSE	FALSE	FALSE	FALSE	FALSE	FALSE	TRUE	TRUE
"No risks involved"	FALSE	FALSE	FALSE	TRUE	FALSE	FALSE	FALSE	FALSE	TRUE	FALSE	FALSE	FALSE	FALSE	FALSE	FALSE	FALSE	FALSE	FALSE	FALSE	FALSE	FALSE	FALSE	FALSE	FALSE	FALSE	FALSE	FALSE	TRUE	FALSE	FALSE	FALSE	TRUE	TRUE	TRUE	TRUE	FALSE	TRUE	TRUE	TRUE	FALSE	TRUE	FALSE	FALSE	FALSE	FALSE	FALSE	FALSE	FALSE	FALSE	FALSE	FALSE
If claims of efficacy used, is there data to back it up?	FALSE	FALSE	FALSE	FALSE	FALSE	FALSE	FALSE	FALSE	FALSE	FALSE	FALSE	FALSE	FALSE	FALSE	FALSE	FALSE	FALSE	FALSE	FALSE	FALSE	FALSE	FALSE	FALSE	FALSE	FALSE	FALSE	FALSE	FALSE	FALSE	FALSE	FALSE	FALSE	FALSE	FALSE	FALSE	FALSE	FALSE	FALSE	FALSE	FALSE	FALSE	FALSE	FALSE	FALSE	FALSE	FALSE	FALSE	FALSE	FALSE	FALSE	FALSE
Use of medical jargon?	FALSE	FALSE	FALSE	FALSE	FALSE	FALSE	FALSE	FALSE	FALSE	FALSE	FALSE	FALSE	FALSE	FALSE	FALSE	FALSE	FALSE	FALSE	FALSE	FALSE	FALSE	FALSE	FALSE	FALSE	FALSE	FALSE	FALSE	FALSE	FALSE	FALSE	FALSE	FALSE	FALSE	FALSE	FALSE	FALSE	FALSE	FALSE	FALSE	FALSE	FALSE	FALSE	FALSE	FALSE	FALSE	FALSE	FALSE	FALSE	FALSE	FALSE	FALSE
View count May 3, 2024	116000	3000	4000	501000	8000	20000	1000	6000	25000	1000	200000	9000	5000	413000	4000	2000	3000	3000	4000	4000	1000	177000	1000	5000	3000	3000	2000	501000	509000	405000	385000	1100000	323000	355000	294000	591000	121000	202000	118000	102000	43000	23000	2000	34000	75000	81000	8000	230000	1700000	N/A	49000
Link	https://library.tiktok.com/ads/detail/?ad_id=1747106845002753	https://library.tiktok.com/ads/detail/?ad_id=1747105573010482	https://library.tiktok.com/ads/detail/?ad_id=1747106915668002	https://library.tiktok.com/ads/detail/?ad_id=1747105512022018	https://library.tiktok.com/ads/detail/?ad_id=1745754621648945	https://library.tiktok.com/ads/detail/?ad_id=1745674213353521	https://library.tiktok.com/ads/detail/?ad_id=1745674216177666	https://library.tiktok.com/ads/detail/?ad_id=1745755692057650	https://library.tiktok.com/ads/detail/?ad_id=1748399617493009	https://library.tiktok.com/ads/detail/?ad_id=1748401420972081	https://library.tiktok.com/ads/detail/?ad_id=1749036557971505	https://library.tiktok.com/ads/detail/?ad_id=1749666863944722	https://library.tiktok.com/ads/detail/?ad_id=1752374302956577	https://library.tiktok.com/ads/detail/?ad_id=1752938430837794	https://library.tiktok.com/ads/detail/?ad_id=1752938799834129	https://library.tiktok.com/ads/detail/?ad_id=1752938799831041	https://library.tiktok.com/ads/detail/?ad_id=1751573956024370	Ad details (tiktok.com)	https://library.tiktok.com/ads/detail/?ad_id=1751022137507890	https://library.tiktok.com/ads/detail/?ad_id=1752938810889250	https://library.tiktok.com/ads/detail/?ad_id=1754649082852385	https://library.tiktok.com/ads/detail/?ad_id=1761880997572609	https://library.tiktok.com/ads/detail/?ad_id=1759267614641185	https://library.tiktok.com/ads/detail/?ad_id=1758715127317521	https://library.tiktok.com/ads/detail/?ad_id=1758071657607170	https://library.tiktok.com/ads/detail/?ad_id=1757374701763617	https://library.tiktok.com/ads/detail/?ad_id=1761709977216050	Ad details (tiktok.com)	https://library.tiktok.com/ads/detail/?ad_id=1762978818560001	https://library.tiktok.com/ads/detail/?ad_id=1761882702113826	https://library.tiktok.com/ads/detail/?ad_id=1757725918047265	https://library.tiktok.com/ads/detail/?ad_id=1775767529318402	https://library.tiktok.com/ads/detail/?ad_id=1764792366594097	https://library.tiktok.com/ads/detail/?ad_id=1774942959084545	Ad details (tiktok.com)	https://library.tiktok.com/ads/detail/?ad_id=1766220622016562	https://library.tiktok.com/ads/detail/?ad_id=1776316943323137	https://library.tiktok.com/ads/detail/?ad_id=1775767529315330	https://library.tiktok.com/ads/detail/?ad_id=1776316943324161	https://library.tiktok.com/ads/detail/?ad_id=1777473615177730	https://library.tiktok.com/ads/detail/?ad_id=1776316943325185	https://library.tiktok.com/ads/detail/?ad_id=1778936706765858	https://library.tiktok.com/ads/detail/?ad_id=1774912768928785	https://library.tiktok.com/ads/detail/?ad_id=1777474630356018	https://library.tiktok.com/ads/detail/?ad_id=1777096495201298	https://library.tiktok.com/ads/detail/?ad_id=1774495736650801	https://library.tiktok.com/ads/detail/?ad_id=1771936498100258	https://library.tiktok.com/ads/detail/?ad_id=1770487460400177	https://library.tiktok.com/ads/detail/?ad_id=1767850768803857	Ad details (tiktok.com)	Ad details (tiktok.com)
Transcript	I don’t know if you guys have seen this, but if you want to get rid of your glasses forever, then you need to know that we are actually looking for people for a free laser eye consultation. Find out if you are eligible, and you can get rid of glasses and contact lenses forever	If you hate wearing glasses, don't skip this video. Each year, millions of people get 20/20 vision or better with laser eye surgery. You don't have to waste money on glasses or contact lenses ever again. You could be eligible for laser eye surgery. Find out today by clicking the link below the video. It doesn't matter what your current prescription is. Laser eye surgery can help with long-sightedness, short-sightedness, and even astigmatism. Go ahead and click the button below this video to book a free consultation	N/A (music playing)	If you are tired of wearing glasses, don’t skip this video. Laser eye surgery can correct short-sightedness, long-sightedness, and even astigmatism. The only question you will ask is "why didn’t I do it sooner?" You can get freedom from glasses and contact lenses, saving you money and hassle. And best of all, with advanced technology, most patients return to work in 48 hours. And financing options mean it is really accessible. Follow thousands of people in the UK who booked their consultation by clicking the link below today	N/A (music)	Hate wearing glasses? Keep watching to find out how you can get rid of them forever. Not being able to see properly without glasses or contacts can be frustrating. Now, millions of people every year are getting their 20/20 vision back through laser eye surgery. It’s safe, and you could start to see improvements just 48 hours after surgery. Laser eye surgery is one of the easiest and most cost-effective ways to correct your vision long term. People across the country have chosen to improve their vision with laser eye surgery, and now you can too	If you hate wearing glasses, don’t skip this video. Book a laser eye surgery consultation today by clicking the ad. Laser eye surgery is more than 99% effective and only takes about 2 minutes. That means you won’t even need someone to look after the dog while you are getting 20/20 vision. [Brand name] have helped thousands of people in the UK get 20/20 vision with laser eye surgery. So what are you waiting for? Click the ad to talk to a specialist today	Hey guys, if you want to ditch your glasses, you need to see this. We are looking for 300 people for a free laser eye consultation. Get rid of those annoying glasses and contact lenses forever	If you are tired of wearing glasses, don’t skip this video. Laser eye surgery can correct short-sightedness, long-sightedness, and even astigmatism. The only question you will ask is why didn’t I do it sooner? You can get freedom from glasses and contact lenses, saving you money and hassle. And best of all, with advanced technology, most patients return to work in 48 hours. And financing options mean it is really accessible. Follow thousands of people in the UK who booked their consultation by clicking the link below today	If you hate wearing glasses, don’t skip this video. Each year, millions of people get 20/20 vision or better with laser eye surgery. You don’t have to waste money on glasses or contact lenses ever again. You could be eligible for laser eye surgery. Find out today by clicking the link below the video	I don’t know if you guys have seen this, but if you want to get rid of glasses forever, then you need to know that we are actually looking for people for a free laser eye consultation. Find out if you are eligible, and you can get rid of glasses and contact lenses forever	I don’t know if you guys have seen this, but if you want to get rid of glasses forever, then you need to know that we are actually looking for people for a free laser eye consultation. Find out if you are eligible, and you can get rid of glasses and contact lenses forever	Something you have to know before going through the LASIK procedure: How much do you spend on glasses every year? The LASIK procedure actually saves you money. 90% of people going through LASIK procedures are receiving the best results. Special discounts for New Year’s. Swipe left to see prices	Hey guys, we are looking for 300 people for a free laser eye consultation. Each year, millions of people get 20/20 vision or better with laser eye surgery. You don’t have to waste money on glasses or contact lenses ever again. You could be eligible for laser eye surgery. It doesn’t matter what your current prescription is. Laser eye surgery can help with long-sightedness, short-sightedness, and even astigmatism. Go ahead and click the button below this video to book a free consultation	I don’t know if you guys have seen this, but if you want to get rid of glasses forever, then you need to know that we are actually looking for people for a free laser eye consultation. Find out if you are eligible, and you can get rid of glasses and contact lenses forever	Hey guys, I just had my laser eye surgery consultation. I am really excited to take my next step and start seeing clearly without using these	The best thing about having laser eye surgery is not having to think about seeing. The surgery itself took minutes. I was actually a bit disappointed. I was telling everyone I was going for this big operation, but it was done so quickly, you barely even realize. Going into the tube and being able to see clearly the next train on the signal—that was sort of a eureka moment for me where I realized "oh my god, I can see." I'd definitely recommend it to friends and family. Having laser eye surgery has improved my quality of life	One thing everyone has to know before going through the LASIK procedure: Struggling with wearing glasses all day? No need to suffer anymore. 95% of people going through LASIK procedures are happy with the results Special discounts for New Year’s. Swipe left to see more	If you hate wearing glasses, don’t skip this video. Each year, millions of people get 20/20 vision or better with laser eye surgery. You don’t have to waste money on glasses or contact lenses ever again. You could be eligible for laser eye surgery. Find out today by clicking the link below the video	Hey guys, do you want to get rid of your glasses? We are looking for 300 people for a free laser eye consultation. So if you want to get rid of glasses forever, tap to see if you are eligible today	Each year, millions of people get 20/20 vision or better with laser eye surgery. You don’t have to waste money on glasses or contact lenses ever again. You could be eligible for laser eye surgery. Find out today by clicking the link below the video. Laser eye surgery can help with short-sightedness, long-sightedness, and even astigmatism. Go ahead and click the button below this video to book a free consultation	Considering laser eye surgery? Don’t wait. Are you tired of wearing glasses or contacts? Laser eye surgery may be the solution for you. Our state-of-the-art technology and experienced surgeons can correct short-sightedness, long-sightedness, and even astigmatism in just a few short minutes. And because the procedure is quick and painless, you can get back to your normal activities right away. Don’t wait any longer; book your free consultation today	Are you tired of wearing glasses and contacts but can't see without them? Well, there’s good news: with LASIK eye surgery, you can achieve crystal-clear vision without glasses or contacts. Say goodbye to glasses, say goodbye to contacts, and say hello to 20/20 vision. Click below to see and live better with LASIK today	Hey, guys. Don’t know if you have seen this, but if you want to get rid of glasses forever, then you need to know that we are actually looking for people for a free laser eye consultation. Check if you are eligible for a free laser eye consultation worth £880 by filling out this quick 60-second form and matching it to top-rated laser eye clinics near you. Go ahead and click the button below to book a free consultation	Hey guys, I don’t know if you have seen this yet, but if you are looking to get rid of glasses forever, then you need to know that we are looking for people for a free laser eye consultation. Find out if you are eligible and get rid of contact lenses and glasses forever	Hey guys, if you are tired of wearing glasses and want to ditch your contact lenses, then you need to know that we're looking for people for a free laser eye consultation. All you need to do is first complete a short form. Then you will receive a call from a laser eye specialist, who can give you a free consultation at a clinic near you as soon as you can make it. Don’t wait any longer; tap below to learn more	N/A (Removed due to terms of service violation)	If you are tired of wearing glasses, don’t skip this video. Laser eye surgery can correct short-sightedness, long-sightedness, and even astigmatism. The only question you will ask is "why didn’t I do it sooner?" You can get freedom from glasses and contact lenses, saving you money and hassle. And best of all, with advanced technology, most patients return to work in 48 hours. And financing options mean it is really accessible. Follow thousands of people in the UK who booked their consultation by clicking the link below today	We are looking for 300 people for a free laser eye consultation. Want to ditch the glasses forever? By filling out this quick and easy quiz, we can match you with the right laser eye surgery provider for you. Once you’re matched, the procedure is quick and painless and can correct long-sightedness, short-sightedness, and astigmatism that cause your vision trouble. Ditch the glasses forever and tap below to learn more	(No voiceover) Born before 1980? Want to get rid of glasses and contact lenses forever? Then you need to know that we are actually looking for 300 people for a free laser eye surgery consultation (worth £800). Tap below to see if you could be eligible	(No voiceover) If you want to get rid of glasses forever, then you need to know that we’re looking for people for a free laser eye consultation. Find out if you’re eligible and you could get rid of glasses and contact lenses forever. Tap "Learn More" to get started	Did you know you can get your perfect vision back in under 30 minutes? With LASIK eye surgery, you can easily restore your eyesight in no time. It is safe, reliable, and thanks to technology, it has now become much more affordable. No jokes- click the link to see prices	Are you born before 1983? Then you could be eligible for a free laser eye consultation. Laser eye surgery can successfully correct short-sightedness, long-sightedness, and even astigmatism. The only question you will ask is "why didn’t I do it sooner?" You can get freedom from glasses and contact lenses, saving you money and hassle. And best of all, with advanced technology, most patients return to work in 48 hours. Financing options mean it is really accessible. Follow thousands of people in the UK who booked their consultation by clicking the link below today	If you're tired of wearing glasses, don't skip this video. Laser eye surgery corrects short-sightedness, long-sightedness, and even astigmatism. The only question you will ask is "why didn’t I do it sooner?" You don't have to waste money on glasses or contact lenses ever again	If you're looking to ditch your glasses or contact lenses, you need to see this. Laser eye surgery corrects short-sightedness, long-sightedness, and even astigmatism. The only question you will ask is "why didn’t I do it sooner?" So if you're fed up with using glasses or contact lenses, follow thousands of people who book their free consultation in the UK by clicking the link below the video today	I don’t know if you guys have seen this, but if you want to get rid of your glasses forever, then you need to know that we are actually looking for people for a free laser eye consultation. Find out if you are eligible, and you can get rid of glasses and contact lenses forever	Did you know you can get your perfect vision back in under 30 minutes? With LASIK eye surgery, you can easily restore your eyesight in no time. It is safe, reliable, and, thanks to technology, it has now become much more affordable. No jokes-click the link to see prices	Did you know you can get your perfect vision back in under 30 minutes? With LASIK eye surgery, you can easily restore your eyesight in no time. It is safe, reliable and thanks to technology it now became much more affordable. No jokes-click the link to see prices	Did you know you can get your perfect vision back in under 30 minutes? With LASIK eye surgery, you can easily restore your eyesight in no time. It is safe, reliable and thanks to technology it now became much more affordable. No jokes- click the link to see prices	We are looking for people from the U K who are interested in our revolutionary laser eye surgery. Tap below to find out more	Did you know you can get your perfect vision back in under 30 minutes? With LASIK eye surgery, you can easily restore your eyesight in no time. It is safe, reliable and thanks to technology it now became much more affordable. No jokes- click the link to see prices	Patient's reaction after LASIK. LASIK eye surgeries have never been so cheap. Everyone can afford it. Swipe left to see prices	Did you know that eye correction used to cost thousands of dollars? Now thanks to technology and competition, eye correction is much more affordable and can go as low as hundreds of dollars. So if you are worried about how much it costs, don't worry. This site can show you the most affordable places to get eye correction near you. Click the link to see prices	We are looking for people from the UK who are interested in our revolutionary laser eye surgery. Tap below to find out more	We are looking for people from the UK who are interested in our revolutionary laser eye surgery. Tap below to find out more	We are looking for people for a free laser eye consultation. The optometrist was super helpful. I got a really in-depth eye test and a quote for my procedure. Tap below to find out more	Hi guys, now i don't know whether it is a well known secret or not, but you can get rid of your glasses forever by getting a free laser eye consultation. Now i only just found out about this, and i am going to mine today. So come along. right guys, so i just left the surgery, and the best thing about this consultation was it's completely free. I am under no obligation to go back but i reckon I will. Stay tuned to find out how the surgery goes. Click below to find out more	If you want to get rid of your glasses forever, then you need to know that we're looking for people for a free laser eye consultation. Find out if you're eligible and you could get rid of glasses and contact lenses forever. All you have to do is click on this ad, fill out your details on this landing page and you'll get booked in for a consultation in no time. It's that easy	I don't know if you guys have seen this but if you want to get rid of your glasses forever, then you need to know that we are looking for people for a free laser eye consultation. Tap below to see if you are eligible	N/A (Removed due to terms of service violation)	Always wanted to get LASIK eye surgery? Here's your sign to get it. It only took 15 minutes and i didn't feel a thing. Swipe left to see prices

## Discussion

Refractive surgery advertising on social media platforms has significant viewership and the potential to impact decision-making for prospective patients. When used effectively, advertising can be a positive influence by disseminating accurate information, dispelling common misconceptions, and highlighting potential hazards or unknown risks. Properly utilized, advertisements can be a powerful tool for improving patient health and quality of life.

In all the advertisements we analyzed, we did not find a single one that included common side effects or complications associated with refractive surgery. Common side effects include dry eyes, glare, diplopia, and under/overcorrections [14]. Additionally, several advertisements described refractive surgery as "safe" or "safer than ever," which may give a false impression of absolute safety and zero risk to potential patients. Some advertisements went further, making potentially hazardous and sensationalized claims such as "the only regret you will have is why didn’t I do this sooner," "refractive surgery actually saves you money on glasses and contacts," and "get rid of glasses forever." These claims disregard the potential for complications and may inappropriately incentivize patients to undertake a procedure that while beneficial, carries financial and medical risks. Moreover, if refractive surgery is performed early in life, the claim of not needing glasses in the future may be misleading due to the development of presbyopia [15].

While claims of efficacy and low risk associated with refractive surgery may be justified given the overall low risk of the procedure, it is essential to provide potential patients with comprehensive information about their options. We found that only 10/51 (20%) of advertisements specified a particular surgical technique (LASIK), and 12/51 (24%) mentioned that all three refractive errors (myopia, hyperopia, and astigmatism) were correctable. Additionally, some advertisements had the potential to mislead patients by suggesting that refractive surgery could address all vision-related issues, such as age-related macular degeneration, cataracts, and glaucoma, which it cannot.

As it stands, many refractive surgery advertisements are likely unvetted due to the difficulty in searching through advertising content on "TikTok" and the lack of access to advertising content on other social media platforms such as "Instagram," "Facebook," "Twitter," and "Snapchat." When asked for a comment, the ASA stated: “The Advertising Standards Authority (ASA) is the UK’s independent advertising regulator. The ASA makes sure ads across UK media stick to the advertising rules (the Advertising Codes). The Advertising Codes require that advertisers hold evidence to prove the claims that they make before they are published or aired. We expect all advertisers to follow the rules when creating their ad campaigns. The Committee of Advertising Practice provides a range of advice, guidance and training to help businesses to get their ads right. For more information about the ASA or CAP, go to www.asa.org.uk.”

Limitations

While gathering our data through "TikTok," we encountered several limitations. We used the main advertisement center for "TikTok" to collect advertisements. Initially, we planned to investigate advertisements on "TikTok," "Twitter (X)," "Snapchat," "YouTube," "Instagram," and "Facebook," as these are the six largest social media networks in terms of active users. However, we faced several challenges. Firstly, "Twitter (X)" and "Snapchat" do not offer a public advertisement library on their platforms. Secondly, while "YouTube" provides a public advertisement library, it only lists advertisements in the form of endorsements reported by video creators and does not allow us to search for advertisements appearing separately from videos. Thirdly, although "Instagram" and "Facebook" also offer public libraries, we found very few unique advertisements; most were duplicates with minor variations based on targeted geographical locations within the UK. Finally, when "Instagram" advertisements included online website redirects, we were unable to locate the stated website links through the "Instagram" advertising library, preventing us from verifying the accuracy of the information or determining if the advertisements were still active.

Furthermore, not all advertisements on the "TikTok" platform are indexed in the advertisement library. This might be because these videos are not marked by "TikTok" as advertisements or because the accounts creating the advertisements are not classified as advertisers. In some cases, certain "TikTok" users with large followings may include paid endorsements within their videos. These endorsements might not be flagged by "TikTok" as advertisements, leading them to be excluded from the main library. It is likely impossible to index all potential advertisements on a social media platform using conventional methods, as there is a reliance on both users and social media platform providers to classify a video as an advertisement or include a paid promotion. Additionally, it is unclear how "TikTok" links each advertisement to the respective keywords searched in the advertisement library. Advertisements may have been missed due to poor keyword associations. Another minor limitation we encountered involved the search function: we were unable to use filters such as “AND” and “OR,” which significantly extended the time needed to find and gather relevant advertisements on the platform.

Recommendations

For Patients

Social media users should be aware that the information in the advertisements they see may not always be accurate. Specifically, their understanding of how refractive surgery is performed, the differences between available procedures, and the potential long-term and adverse effects of these procedures may be limited.

For Doctors

Ophthalmologists, healthcare professionals, and healthcare providers should be aware that content produced by advertising companies to promote refractive error correction services on social media platforms may not comply with RCOphth guidelines. Patients who have seen such advertisements on social media may require a more thorough and intensive consent process.

For Social Media Platforms

Social media platforms, including "Instagram," "Facebook," "Twitter," and "Snapchat," should enhance their advertising search engines to improve the vetting of advertisements on their platforms. This would help ensure that advertisements meet professional standards and are safe, particularly those involving medical procedures where there is potential for significant physical, mental, and financial risk to potential patients. This approach should be extended to include not just refractive surgery but all types of medical procedures.

To improve access to these advertisements, social media platforms could establish a separate, searchable library specifically for health products and procedures. The search function should include basic search modifiers such as “AND” and “OR,” as well as filters like “date,” “advertiser,” and “age demographic reached.”

Aside from manual indexing and keyword association in an advertising library, employing artificial intelligence (AI)-based speech-to-text and natural language analysis could help ensure that a greater number of potential advertisements on a social media platform are accounted for compared to manual labelling alone. However, training such AI systems is likely to be an expensive and time-consuming process at present.

Regular auditing of advertisements should be conducted by a third party. Advertisements that have been audited could receive a boost in visibility on the main social media platform, such as verification “checkmarks” to indicate that the information provided has been vetted and is of high quality. This would help social media users distinguish reputable advertisements from those of unknown quality, thereby enhancing patient autonomy and incentivizing advertisers to proactively ensure their advertisements are correctly labelled and audited. Although this process may take several years to fully implement, it would significantly improve the quality of refractive surgery advertising and health advertising overall.

For Regulators

While the UK ASA regulates advertisements for certain medical fields, specific rules for refractive surgery are not covered under the ASA's nonbroadcast code of marketing communications. Although the nonbroadcast code states that all “marketing communications should be legal, decent, honest, and truthful” [16], advertisements on social media platforms are generally not covered by this code. We believe the ASA should take a more active role in regulating refractive surgery advertisements, particularly for "marketers with a UK-registered company address," “marketing communications appearing on websites with a “.uk” top-level domain,” and “paid-for marketing communications from or by marketers targeting people in the UK” [17]. The RCOphth should collaborate with the ASA and social media platforms to raise awareness of its guidelines and support the regular review of social media advertising content.

## Conclusions

In conclusion, our findings indicate that current refractive surgery advertising practices on social media platforms do not meet the standards set by the RCOphth. We identified issues such as a lack of pertinent information, misleading claims, and potentially hazardous content for patients. We believe that a collaborative approach involving social media platforms, the ASA, and the RCOphth is necessary to address these issues and establish higher advertising standards that empower patients with comprehensive information. In this paper, we have highlighted the impact of refractive surgery advertising on social media users and proposed changes to improve the content of advertisements, the functionality of social media advertisement libraries, and the vetting process for refractive surgery advertising.
